# Supplementary material for: Content-rich biological network constructed by mining PubMed abstracts
Source: BMC Bioinformatics. 2004 Oct 8;5:147. doi: 10.1186/1471-2105-5-147 (PMC528731; doi:10.1186/1471-2105-5-147)
Supplement: Additional File 2 — The original results of the above study (non-essential files are deleted to keep the file size under the limit set by BMC bioinformatics). [file 1471-2105-5-147-S2.bz2 › chilibotAdditionalFile2/dip05/339ID1280226E4221/html/TNFSF5_TNFRSF5.html]

 


 **TNFSF5** and **TNFRSF5** 
  
Found 2289 abstracts in PubMed, retrieved 05.  
 

 What does Google say? 
 PDF only 
| .edu only 

---

**Interactive relationship** (e.g. stimulation, inhibition, etc)

**Neutral relationship**- These patients presented opportunistic infections as observed in X linked HIGM, suggesting that the CD40  [ **TNFRSF5** ]  CD40  [ **TNFRSF5** ]  ligand  [ **TNFSF5** ]  interaction is important for promoting T cell mediated immunity.  Ref: 12893749 BloodBlood,

**Non-interactive relationship** (e.g. studied together, co-existance, homology, etc.)

- Furthermore, both hCD100 Fc and anti human CD72 agonistic mAb induce the production of the pro inflammatory cytokines tumor necrosis factor  [ **TNFSF5** ]  alpha, IL 6 and IL 8, even in the absence of anti CD40  [ **TNFRSF5** ]  mAb.  Ref: 12882840 Int ImmunolInt Immunol,
- In this study, we investigate whether A20 a gene we have shown to be antiinflammatory and antiapoptotic in ECs can protect from CD40  [ **TNFRSF5** ]  CD40L  [ **TNFSF5** ]  mediated EC activation.  Ref: 12885753 CirculationCirculation,
- A20 Protects From CD40  [ **TNFRSF5** ]  CD40  [ **TNFRSF5** ]  Ligand  [ **TNFSF5** ]  Mediated Endothelial Cell Activation and Apoptosis.  Ref: 12885753 CirculationCirculation,
- BACKGROUND CD40  [ **TNFRSF5** ]  CD40  [ **TNFRSF5** ]  ligand  [ **TNFSF5** ]  CD40L  [ **TNFSF5** ]  signaling is a potent activator of endothelial cells ECs and promoter of atherosclerosis.  Ref: 12885753 CirculationCirculation,
- Altogether, these data show that in contrast to CD40  [ **TNFRSF5** ]  ligand  [ **TNFSF5** ] , which induced LC maturation even in presence of TGF beta1, nonspecific proinflammatory factors such as TNF alpha, IL 1 or LPS, essentially induced immature LC generation, and little cell activation in the presence of TGF beta1.  Ref: 12884872 Eur J Immunol, 2003
- When added to GM CSF, IL 4 and TGF beta1 containing cultures, LPS or IL 1beta also induced significant numbers of Langerin CD83 immature cells displaying a low allostimulatory activity, while CD40  [ **TNFRSF5** ]  ligand  [ **TNFSF5** ]  largely promoted highly allostimulatory Langerin CD83 cells.  Ref: 12884872 Eur J Immunol, 2003
- CONCLUSIONS A20 works at multiple levels to protect ECs from CD40  [ **TNFRSF5** ]  CD40L  [ **TNFSF5** ]  mediated activation and apoptosis.  Ref: 12885753 CirculationCirculation, 2003
